# Tetracycline-Containing MCM-41 Mesoporous Silica Nanoparticles for the Treatment of *Escherichia coli*

**DOI:** 10.3390/molecules201119650

**Published:** 2015-10-30

**Authors:** Bhuvaneswari Koneru, Yi Shi, Yu-Chieh Wang, Sai H. Chavala, Michael L. Miller, Brittany Holbert, Maricar Conson, Aiguo Ni, Anthony J. Di Pasqua

**Affiliations:** 1 Department of Pharmaceutical Sciences, University of North Texas System College of Pharmacy, University of North Texas Health Science Center, 3500 Camp Bowie Blvd., Fort Worth, TX 76107, USA; E-Mails: bhuvaneswari.koneru@live.unthsc.edu (B.K.); yi.shi@unthsc.edu (Y.S.); yu-chieh.wang@unthsc.edu (Y.-C.W.); bholbe02@g.uafs.edu (B.H.); maricar.conson@my.unthsc.edu (M.C.); 2 North Texas Eye Research Institute, University of North Texas Health Science Center, 3500 Camp Bowie Blvd., Fort Worth, TX 76107, USA; E-Mail: sai.chavala@unthsc.edu; 3 Department of Chemistry, Creighton University, 2500 California Plaza, Omaha, NE 68178, USA; E-Mail: michaelmiller@creighton.edu; 4 Department of Cell Biology and Immunology, Graduate School of Biomedical Sciences, University of North Texas Health Science Center, 3500 Camp Bowie Blvd., Fort Worth, TX 76107, USA; E-Mail: aiguo.ni@unthsc.edu

**Keywords:** tetracycline, MCM-41, *E. coli*, controlled drug release

## Abstract

Tetracycline (TC) is a well-known broad spectrum antibiotic, which is effective against many Gram positive and Gram negative bacteria. Controlled release nanoparticle formulations of TC have been reported, and could be beneficial for application in the treatment of periodontitis and dental bone infections. Furthermore, TC-controlled transcriptional regulation systems (Tet-on and Tet-off) are useful for controlling transgene expression *in vitro* and *in vivo* for biomedical research purposes; controlled TC release systems could be useful here, as well. Mesoporous silica nanomaterials (MSNs) are widely studied for drug delivery applications; Mobile crystalline material 41 (MCM-41), a type of MSN, has a mesoporous structure with pores forming channels in a hexagonal fashion. We prepared 41 ± 4 and 406 ± 55 nm MCM-41 mesoporous silica nanoparticles with loaded TC for controlled drug release; TC content in the TC-MCM-41 nanoparticles was 18.7% and 17.7% *w*/*w*, respectively. Release of TC from TC-MCM-41 nanoparticles was then measured in phosphate-buffered saline (PBS), pH 7.2, at 37 °C over a period of 5 h. Most antibiotic was released from both over this observation period; however, the majority of TC was released over the first hour. Efficacy of the TC-MCM-41 nanoparticles was then shown to be superior to free TC against *Escherichia coli* (*E. coli*) in culture over a 24 h period, while blank nanoparticles had no effect.

## 1. Introduction

Tetracycline (TC) is a broad spectrum antibiotic which has been used for more than 60 years [1,2], and is on the World Health Organization’s list of most important medications needed in the basic health system [3]. TC is effective against various Gram positive and Gram negative bacteria [2,4], and using controlled release formulations for the delivery of TC could help maintain efficacious levels of the antibiotic in patients; controlled release TC, when given in combination with a traditional treatment, was shown to decrease the recurrence of periodontic disease in adult patients [5]. TC-controlled transcriptional regulation systems (Tet-on and Tet-off systems) are useful in controlling transgene expression *in vitro* and *in vivo* for biomedical research purposes [6]; controlled release TC formulations that allow for appropriate concentrations of TC to be maintained over long periods of time, after only one administration, would be useful for such systems. Various formulations for controlled release of TC have been reported. For example, Kenawy *et al.* [7], developed an electrospun polymer fiber for controlled release of TC (25% *w*/*w* TC loading), and Govender *et al.* [8] formulated and optimized bioadhesive controlled release TC microspheres (10% *w*/*w* TC loading). Luginbuehl *et al.* [9], reported controlled release of TC from biodegradable β-tricalcium phosphate composites (2.2% ± 0.2% and 2.9% ± 0.2% *w*/*w* TC loading when using fast and slow degrading polymers, respectively).

Mesoporous silica nanomaterials (MSNs) are widely studied for drug delivery applications [10,11,12,13]. Mobile crystalline material 41 (MCM-41), a type of MSN, was first reported by Beck *et al.* in 1992 [14]. MCM-41 has a mesoporous structure with pores forming channels in a hexagonal fashion [10,15]. Its tunable intrinsic properties like pore size, particle size and large surface area make it an ideal host for various molecules. Therapeutic formulations of various compounds like anti-inflammatory agents (*i.e.*, naproxen), antibiotics (*i.e.*, vancomycin) and anticancer agents (*i.e.*, carboplatin) have been developed using MCM-41 formulations [12,13,16], and MCM-41 contributes to controlled drug release [17,18,19,20]. MCM-41 was previously shown to be effective in adsorbing TC from aqueous solutions to avoid environmental pollution [21,22,23]. TC-loaded 100 nm MCM-41 spheres (12.7% *w*/*w* TC loading) were previously prepared and release of TC studied [24]. Hashemikia *et al.* [25] reported adsorption and controlled release of TC in SBA-15 type mesoporous silica nanoparticles (42.3% *w*/*w* TC loading).

A study on the effect of particle size of MSNs on cellular uptake *in vitro* showed that 30 and 50 nm MSNs were taken up by cells more so than >100 nm MSNs [26]. However, MSNs sized 400 nm have been shown to have therapeutic potential in *in vivo* models [27]. To explore controlled release of TC using MSNs of different sizes, we prepared two different sized TC-containing MCM-41 type MSNs (41 ± 4 nm and 406 ± 55 nm) and investigated their *in vitro* release profiles in a biological relevant buffer. We then tested their efficacy against *Escherichia coli* (*E. coli*) in culture.

## 2. Results and Discussion

### 2.1. Preparation of TC-Containing MCM-41 MSNs and Transmission Electron Microscopy (TEM)

**Figure 1:**
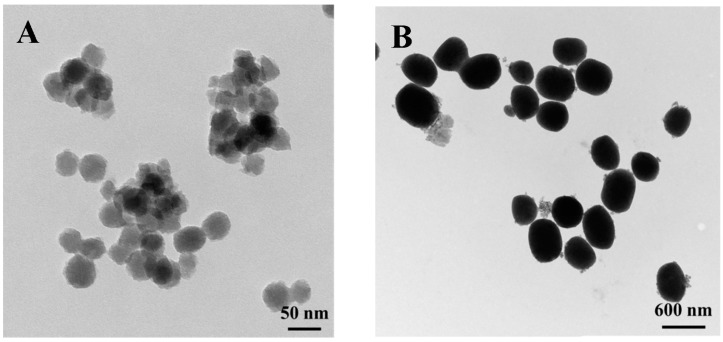
TEM images of tetracycline-containing MCM-41 nanoparticles. (**A**) TC-MCM-41A (41 ± 4 nm) and (**B**) TC-MCM-41B (406 ± 55 nm).

MCM-41 nanoparticles of two sizes (MCM-41A and MCM-41B) were synthesized following published procedures [27,28]. TC was loaded by exposing 20 mg of either MCM-41A or MCM-41B to a solution containing 10 mg/mL of TC hydrochloride in Milli-Q water (EMD Millipore; Billerica, MA, USA) and the mixture was stirred vigorously for 24 h at room temperature (r.t.). The TC-loaded nanoparticles (TC-MCM-41A and TC-MCM-41B) were characterized using transmission electron microscopy (TEM; Zeiss EM 910 transmission electron microscope; Zeiss, Jena, Germany). Particle size was measured from TEM images using ImageJ software (1.47, National Institute of Health, Bethesda, MD, USA). TC-MCM-41A had a particle size of 41 ± 4 nm (Figure 1A) and TC-MCM-41B has a particle size of 406 ± 55 nm (Figure 1B).

**Figure 2:**
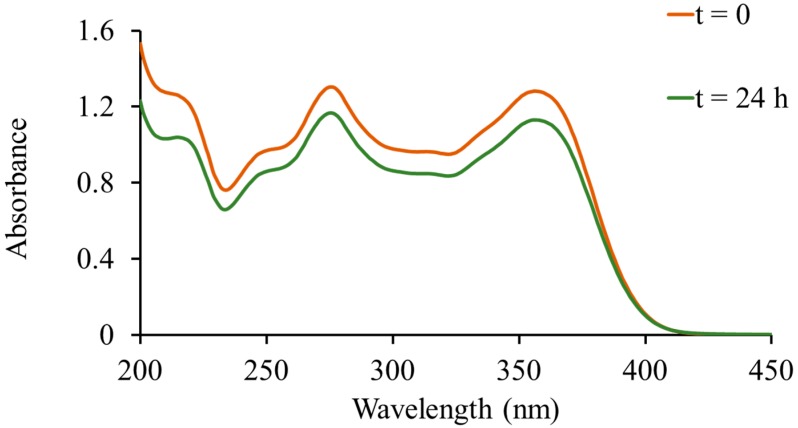
UV-Vis spectra of tetracycline at 0 and 24 h. A slight downward shift in the spectra was observed at 24 h.

**Figure 3:**
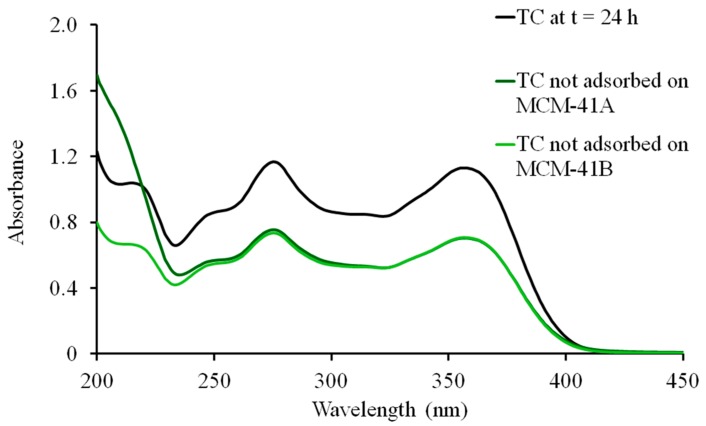
Adsorption of tetracycline (TC) by MCM-41A and MCM-41B. UV-Vis spectra of TC control at 24 h and TC not adsorbed by MCM-41A (green) and MCM-41B (light green). Percent drug adsorption was measured by comparing absorbance at 275 nm.

UV-Vis spectra of TC in Milli-Q water (10 mg/mL; control) were collected (Cary 60 UV-vis spectrophotometer; Agilent Technologies, Santa Clara, CA, USA) at 0 and 24 h (Figure 2). A slight downward shift in the TC spectrum was observed after 24 h, most likely due to the instability of TC in aqueous solution [29]. After loading the MSNs with TC, the supernatants from all washes were collected and the UV-vis spectra obtained (Figure 3). Percent loading of TC was calculated by comparing the absorption at λ = 275 nm of the supernatants to that of the 24 h TC control. The weight percent of TC in TC-MCM-41A and TC-MCM-41B formulations were 18.7% and 17.7% *w*/*w*, respectively. Thus, drug loading is similar to that achieved by Lin *et al.* (12.7% *w*/*w* TC) using 100 nm MCM-41 MSNs [24]. When impregnated with lanthanum (La), MCM-41 was shown to adsorb TC more efficiently from aqueous samples in environmental studies; TC loading increased from approximately 3% to 20% [21]. When using SBA-15 to load drug, TC content was 42.3% *w*/*w* [25]. Variations in loading are most likely due to differences in physicochemical properties of the materials; however, how these differences affect biocompatibility of the nanoparticle need be considered.

### 2.2. In Vitro Drug Release

**Figure 4:**
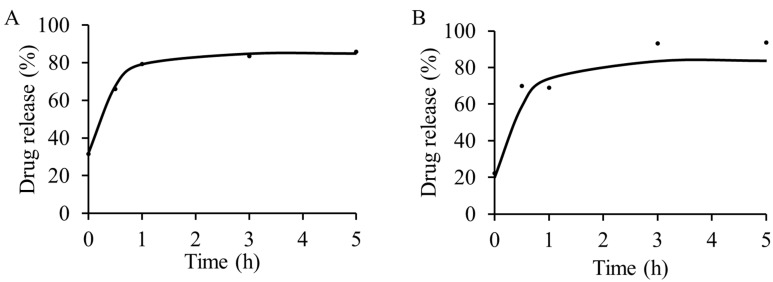
*In vitro* release of tetracycline from (**A**) TC-MCM-41A and (**B**) TC-MCM-41B, determined using UV-vis spectroscopy. Most drug was released by 5 h. All studies were carried out in PBS, pH 7.2, at 37 °C.

The *in vitro* release of TC in phosphate buffered saline (PBS; pH 7–7.2) at 37 °C was measured using UV-vis spectroscopy. Release was determined at 0, 0.5, 1, 3 and 5 h for both TC-MCM-41A and TC-MCM-41B (Figure 4). A burst release was observed in both formulations at *t* = 0 and most drug was released by the end of the 5 h observation period (86% and 94% for TC-MCM-41A and TC-MCM-41B, respectively), although most TC was released over the first hour. The drug release from our formulations in PBS is faster, compared to the formulation by Lin *et al.* [24] in simulated biological fluid (SBF; pH 7.4) at 37 °C; after five days 41.9% TC was released by the 100 nm MCM-41 in SBF. It is known that differences in pore size can affect drug release from MSNs [30,31], and this could be a contributing factor here. Drug release rate can also be controlled by functionalization of MSNs. For example, release of vancomycin from CdS capped MCM-41 type MSNs was shown to be extended for up to 3 days [13]. A controlled release of captopril using MCM-41 showed enhanced release profile upon silylation [18]. Amine functionalized SBA-15 type MSNs release of TC was shown to be extended up to 48 h [25]. Thus, drug release could potentially be extended from our materials by surface functionalization.

### 2.3. Antibacterial Activity against E. coli

**Figure 5:**
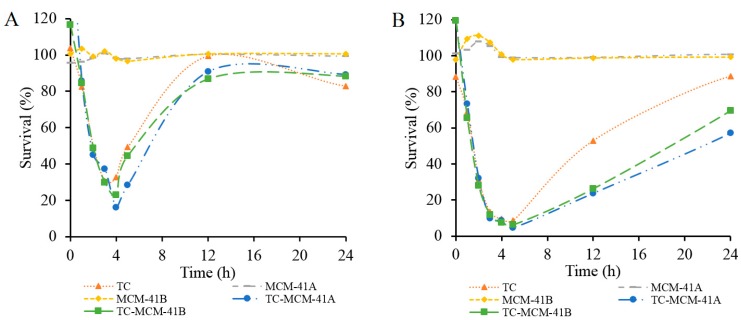
Survival (%) of *E. coli* treated by TC, MCM-41A, MCM-41B, TC-MCM-41A and TC-MCM-41B, determined using UV-vis spectroscopy. (**A**) TC concentration: 0.5 µg/mL and (**B**) TC concentration: 1.0 µg/mL. In each group, the concentration of free TC was the same as the concentration of TC in TC-MCM-41A and TC-MCM-41B, and concentrations of MCM-41A and MCM-41B corresponded to concentrations of TC-MCM-41A and TC-MCM41B, respectively.

The antibacterial activity of prepared TC-MCM-41A and TC-MCM-41B was tested against *E. coli*, and compared with that of free TC and blank MCM-41A and MCM-41B, Figure 5. Two groups with different TC concentrations, 0.5 µg/mL (Group 1, Figure 5A) and 1.0 µg/mL (Group 2, Figure 5B), were investigated. In each group, the concentration of free TC was the same as the concentration of TC in TC-MCM-41A and TC-MCM-41B, and concentrations of MCM-41A and MCM-41B corresponded to concentrations of TC-MCM-41A and TC-MCM-41B, respectively. As illustrated in Figure 5, MCM-41A and MCM-41B did not show any effect on *E. coli* growth in either group. Within 4 h after treatment, free TC and TC-MCM-41A and TC-MCM-41B showed similar inhibition of *E. coli* growth. However, after 4 h and up to 18 h for Group 1 and up to 24 h for Group 2, TC-MCM-41A and TC-MCM-41B both exhibited greater inhibition on bacteria growth than free TC. Percent survival of *E. coli* treated with 10 µg/mL free TC decreased to 3% in 4 h and did not increase with time (data not shown). After 24 h, the *E. coli* with no treatment stopped growing as nutrients were exhausted. Slow release of TC could preserve the efficacy of TC, as not all antibiotic is in the medium at *t* = 0. Thus, differences in efficacy may be due to TC in the pores being less susceptible to degradation in the medium, compared to free TC; however, differences in uptake of free TC and TC-MCM-41A and TC-MCM-41B cannot be discounted. Previously, doxycycline-loaded nanoparticles were shown to improve antibacterial efficacy of doxycycline, and this was attributed to its sustained release profile and prolonged stability of loaded doxycycline compared to free drug [32].

## 3. Experimental Section

### 3.1. General Information

Tetraethoxysilane (TEOS), hexadecyltrimethylammonium bromide (CTAB), sodium hydroxide and TC were purchased from Sigma-Aldrich (St. Louis, MO, USA). Anhydrous ethanol was obtained from Pharmco-AAPER (Belmont, NC, USA). Phosphate buffered saline (0.0067 M; pH 7.0–7.2) was purchased from HyClone laboratories (Logan, UT, USA).

### 3.2. Synthesis of Tetracycline-Containing MCM-41 Nanoparticles

MCM-41A MSNs were prepared following previously published procedures with slight modifications [27,28]. NaOH (7 mL, 0.04 M) was added to H_2_O (480 mL) and the solution was heated to 60 °C. Then, CTAB (2.0 g) was added, followed by TEOS (11.3 mL, 50.6 mmol), while stirring. The mixture was stirred for another 10 min at 60 °C, before filtered using aspiration and the precipitate washed three times with Milli-Q water (20 mL) followed by two additional rinses with absolute ethanol (20 mL). Later, a portion of the moist product (1.0 g) was added to absolute ethanol (150 mL) followed by concentrated HCl (0.5 mL) while stirring at r.t. for 2 h. The resulting white precipitate was vacuum filtered and washed with Milli-Q water (50 mL) followed by ethanol (50 mL) and dried *in vacuo* to yield MCM-41A. MCM-41B was prepared as previously described [27]. Then, MCM-41A or MCM-41B (20 mg) were added to a 1 mL solution containing TC (10 mg/mL, 20.8 mM) in Milli-Q water. The suspension was stirred vigorously for 24 h at r.t. and then centrifuged at 1300× *g* for 20 min. The resulting pellet was washed three times with H_2_O and dried *in vacuo* for 24 h. The supernatants from each step were collected and UV-vis spectra obtained. The dried MSNs were imaged using TEM.

### 3.3. UV-Vis Spectroscopy

For estimating the drug adsorption by the nanoparticles, UV-vis spectroscopy was used. A 10 mg/mL solution of TC in Milli-Q water was prepared and the UV-vis absorption spectra measured at 0 and 24 h. Supernatants from all washes during the preparation of TC-containing MCM-41 nanoparticles were collected and UV-vis spectra recorded. The spectra of TC at 24 h was compared with that of the supernatants to calculate the drug adsorbed on the nanoparticles after 24 h. Percent drug incorporated was determined from the following formula:

(1) $$\mathrm{Drug incorporated}\;(\%)\;=\;\left[\;\frac{\mathrm{Drug incorporated}\;\left(\mathrm{mg}\right)}{\mathrm{Drug}-\mathrm{containing MSNs}\;\left(\mathrm{mg}\right)}\right]\;\times \;100$$

### 3.4. In Vitro Release Studies

All release studies were carried out in PBS, pH (7–7.2), at 37 °C. TC- MCM-41A or TC-MCM-41B was weighed and suspensions of 1 mg/mL in PBS were prepared. The suspensions were then stirred vigorously at 37 °C, and release measured at 0, 0.5, 1, 3 and 5 h from the different suspensions. Samples were removed at their respective time points and centrifuged at 1000× *g* for 10 min at r.t. Supernatant was collected and the UV-vis spectra obtained. The amount of drug (mg) released was calculated by comparing the absorbance at 275 nm to the absorbance of a 10 mg/mL solution of TC in PBS. Percent drug released was calculated from both TC-MCM-41A and TC-MCM-41B using the following formula:

(2) $$\mathrm{Drug release}\;(\%)\;=\;\left[\;\frac{\mathrm{Drug released}\;\left(\mathrm{mg}\right)}{\mathrm{Drug loaded on MSNs}\;\left(\mathrm{at}\;\mathrm{time}\;=\;0,\;\mathrm{mg}\right)}\right]\;\times \;100$$

The obtained data was fit to an exponential regression using Microsoft Excel Solver.

### 3.5. Antibacterial Activity of TC-MCM-41A and TC-MCM-41B against E. coli

The antibacterial activity of prepared TC-MCM-41A and TC-MCM-41B was further tested using *E. coli*, which was cultured in round-bottom culture tubes at 37 °C in a shaker at 250 rpm, with a tube volume to culture volume ratio of 1.75:1. Lennox L broth (LB broth; Research Products International Corp. Mt Prospect, IL, USA) was used as growth medium.

The antibacterial activity was tested at two different concentrations of TC (1) 0.5 μg/mL and (2) 1.0 μg/mL. *E. coli* was pre-cultured in 4 mL medium at conditions mentioned above. From this 800 μL of *E. coli* was taken and inoculated into 250 mL of culture medium. This was further split into different culture tubes and received one of the following treatments. Group 1: (a) blank (no treatment); (b) 0.5 μg/mL TC alone; (c) MCM-41A; (d) MCM-41B; (e) TC-MCM-41A (0.5 μg/mL TC); and (f) TC-MCM-41B (0.5 μg/mL TC). Group 2: (a) blank (no treatment); (b) 1.0 μg/mL TC alone; (c) MCM-41A; (d) MCM-41B; (e) TC-MCM-41A (1.0 μg/mL TC) and (f) TC-MCM-41B (1.0 μg/mL TC) and (g) 10 μg/mL TC (positive control). In each group, the concentration of free TC was the same as the concentration of TC in MCM-41A and MCM-41B, and concentrations of MCM-41A and MCM-41B corresponded to concentrations of TC-MCM-41A and TC-MCM-41B, respectively.

The growth of *E. coli* in all treatment groups was then monitored in terms of change in turbidity at 0, 1, 2, 3, 4, 5, 12, 24, 30, 36, 48, 60 and 72 h by measuring UV-Vis spectra. Absorbance at 600 nm was used to compare the growth inhibition among various treatments [33]. %Survival was calculated using the following equation:

(3) $$\mathrm{Survival}\;(\%)\;=\;\left[\frac{\mathrm{Absorbance of treatment group}\;(\mathrm{at}\;600\;\mathrm{nm})}{\mathrm{Absorbance of no treatment group}\;(\mathrm{at}\;600\;\mathrm{nm})}\right]\;\times \;100$$

## 4. Conclusions

MCM-41 nanoparticles loaded with TC (TC-MCM-41A and TC-MCM-41B) were prepared in two different sizes (41 ± 4 nm and 406 ± 55 nm, respectively); drug adsorption measured using UV-vis spectroscopy was 18.6% and 17.7% *w*/*w* TC, respectively. *In vitro* release studies were performed in PBS, pH 7.2, at 37 °C and the efficacies of TC-MCM-41A and TC-MCM-41B were shown to be superior to free TC *in vivo*, while blank nanoparticles had no effect.
